# Death of Dr. J. Dixon Mann

**Published:** 1912-04-13

**Authors:** 


### Obituary.

### Death of Dr. J. Dixon Mann.

Mr. John Dixon Mann, Professor of Forensic Medicine and Toxicology at Victoria University, Manchester, has died after a short illness. For more than thirty years he was honorary physician at the Salford Royal Hospital, and late Examiner in Forensic Medicine for the three Universities of Oxford, London, and Sheffield. He qualified in 1862 and graduated M.D. at St. Andrews in 1880. Ten years later he took his degree as L.R.C.P. Lond. He was the author of "Forensic Medicine and Toxicology" and many other publications.

